# On Maternal Burnout Assessment Tool validity and reliability: key statistical indicators

**DOI:** 10.1192/j.eurpsy.2026.12205

**Published:** 2026-07-31

**Authors:** E. Polonetskaya, E. Pervichko

**Affiliations:** Faculty of Psychology, Lomonosov Moscow State University, Moscow, Russian Federation

## Abstract

**Introduction:**

Maternal burnout is a widely recognized condition seriously affecting the life of a mother, her child and those around them. The importance of its timely identification, as well as increasing general awareness of the characteristics of this phenomenon, may not be overestimated. As a result, there exists a need for modern instruments for the assessment of the condition in Russian language, which meet up-to-date validity requirements.

**Objectives:**

Our research objective was to create a tool for studying the condition of maternal burnout based on a recent and internationally recognized burnout questionnaire, so that maternal burnout may then be suitably assessed, and potentially compared to work-related burnout in the future.

**Methods:**

A maternal burnout questionnaire in Russian (the Maternal Burnout Assessment Tool, MBAT) has been prepared on the basis of the work-related Burnout Assessment Tool (BAT) questionnaire by replacing the references to work with references to childcare and making other minor adjustments, as required, to appropriately adapt the work-related BAT wording. The surveys were conducted between September 2023 and January 2025. The samples consisted of up to 792 mothers with children up to seven years of age. Additionally, to measure optimism, depressive tendencies and anxiety, LOT-R, BDI and State-Trait Anxiety Inventory by C. Spielberger in their respective Russian adaptations were used.

**Results:**

We have achieved the replication of the original BAT-23 four-factor structure (as supported by confirmatory factor analysis), with the exception of question 13, which has been dropped as not duly fitting the factor structure based on the relevant statistical checks. The resulting model fit indices are as follows (N=792):

**Table 1.** Table 1. Long description.

*χ2*	S-Bχ2*	Df**	RMSEA	CFI	TLI	SRMR
644	556	203	0.052	0.956	0.950	0.037

^*^ Chi-square as adjusted by the Satorra–Bentler scaling factor ^**^ p <0,001

The internal consistency of the MBAT has been supported by the following Cronbach’s alpha coefficients: the composite 0.94 for the total burnout score, 0.90 for the exhaustion subscale, 0.83 for the mental distance subscale, 0.89 for the cognitive impairment subscale and 0.87 for the emotional impairment subscale.

As to the convergent and discriminant validity, it is confirmed by the associations of the total burnout score with, among others, such indicators as: optimism (N=469, r=-0.52, p <0,001), depressive tendencies (N=392, r=0.62, p <0,001), state anxiety (N=356, r=0.61, p <0,001) and trait anxiety (N=356, r=0.57, p <0,001) (in each case using Spearman’s rank correlation coefficient).

**Conclusions:**

In view of the above, the MBAT, being an instrument created on the basis of the BAT model and wording, is a valid and reliable tool for assessing maternal burnout and may be used for research purposes. For the use of this questionnaire in assessing paternal burnout, further research on an adequate sample of fathers needs to be conducted.

**Disclosure of Interest:**

None Declared

## Long descriptions

### Table 1. Long description

The table consists of seven columns and one data row.

Header row from left to right:

- chi-square symbol (chi super 2)

- S-B chi super 2 asterisk

- D f double asterisk

- R M S E A

- C F I

- T L I

- S R M R

Data row values:

- chi super 2: 644

- S-B chi super 2 asterisk: 556

- D f double asterisk: 203

- R M S E A: 0.052

- C F I: 0.956

- T L I: 0.950

- S R M R: 0.037

Footer notes:

- Asterisk: Chi-square as adjusted by the Satorra-Bentler scaling factor.

- Double asterisk: p is less than 0,001.

Navigate back to Table 1..

